# Effect of adding arginine at different concentrations to experimental orthodontic resins: an *in vitro* study

**DOI:** 10.1590/1807-3107bor-2024.vol38.0078

**Published:** 2024-09-02

**Authors:** Ana Lídia Correa SANTOS, Lourenço CORRER-SOBRINHO, Fernanda Midori TSUZUKI, Anália Gabriela FACURY FERRAZ, José Guilherme NEVES, Mário Alexandre Coelho SINHORETI, Eduardo Martinelli FRANCO, Ana Rosa COSTA

**Affiliations:** (a) Fundação Hermínio Ometto, Department of Orthodontics, Araras, SP, Brazil.; (b) Universidade Estadual de Campinas – Unicamp, Piracicaba Dental School, Department of Restorative Dentistry, Piracicaba, SP, Brazil.; (c) Universidade Estadual de Campinas – Unicamp, Piracicaba Dental School, Department of Microbiology, Piracicaba, SP, Brazil.

**Keywords:** Orthodontics, Composite resins, Arginine, Mechanical tests, Streptococcus mutans

## Abstract

The aim of this study was to assess the effect of adding arginine at different concentrations to commercial and experimental orthodontic resins on shear bond strength (SBS), as well as on the antimicrobial activity of arginine against *S. mutans.* Metal brackets were bonded onto the surface of 120 bovine incisors using Transbond, OrthoCem, and an experimental resin (ER), adding 0, 2.5, 5, and 7 wt.% of arginine. The SBS test was performed in deionized water at 37 ºC for 24 h, at 0.5 mm/min. SBS test results were subjected to two-way ANOVA and Tukey’s test (α = 0.05). CFU/mL data (antimicrobial assessment) were assessed by Kruskal-Wallis and Dunn’s tests (α = 0.05). No statistical difference between the resins was observed in untreated groups (p > 0.05). The addition of arginine at 2.5% (27.7 MPa) and 5% (29.0 MPa) increased the SBS of Transbond when compared (p < 0.05) to OrthoCem (18.5 and 15.6 MPa, respectively) and ER (16.3 and 18.1 MPa, respectively). Arginine at 7% improved the SBS of Transbond (24.1 MPa) and ER (21.0 MPa), which was statistically higher (p < 0.05) than OrthoCem (12.6 MPa). OrthoCem did not show a statistically significant difference at the three concentrations of arginine (p > 0.05). The addition of arginine to resins reduced the count of *S. mutans* (p < 0.05). As for ER, all concentrations of arginine significantly decreased CFU/mL (p < 0.05). Among commercial resins, only 7% of arginine significantly reduced CFU/mL. The addition of arginine did not interfere with the bond strength and demonstrated antibacterial activity against *S. mutans.*

## Introduction

Dental caries is one of the most common oral diseases^1-3^ and it is caused by microorganisms present in oral biofilm in a sugar-rich diet and under poor oral hygiene conditions.^1,4,5^Another very common problem in the oral cavity is malocclusion, whose recommended treatment consists in bonding orthodontic brackets to the enamel to correct malpositioned teeth.^6^ Food remains get trapped around the wires and accessories of orthodontic brackets, hindering oral hygiene and causing biofilm accumulation on the tooth structure and changes in the properties of saliva and microbial count.^7-14^ One of the side effects of orthodontic treatment is the development of white spot lesions, with a reported incidence of 32% to 72.9%,^10^ found more frequently at the cervical third and on the margins around the brackets.^10,15^

In addition to the orthodontic appliance and its accessories, the resin used for bonding the brackets may be a predisposing factor for caries, as adhesive failure and excess material may lead to biofilm accumulation.^11,15,16^ Note that the ideal orthodontic bonding material should have a minimum bond strength from 5.9 to 7.8 MPa so it can withstand masticatory forces and the forces applied during the treatment.^14,17,18^

The well-structured organization of the oral biofilm hinders the development of an efficient therapy for dental caries control.^5,18^ The use of fluoride in wearers of orthodontic appliances reduces demineralization.^6,13,14,18,19^ Nevertheless, using dentifrice, gels, varnishes, and fluoride mouthwashes requires patient collaboration and constant reapplication. Another preventive alternative, which does not rely on patient collaboration, is using bonding agents with the addition of fluoride to the bonding materials.^13,14^

The addition of antibacterial properties to orthodontic bonding agents is another way to reduce the development of white spot lesions.^8,9,11,18^ Arginine, increasingly used in dentistry nowadays, is an amino acid present in foods and naturally found in the saliva. It is hydrolyzed by arginine deiminase (ADI), generating ammonia.^2-4,6,20-23^ Ammonia production inhibits dental demineralization, neutralizes acids, and positively affects the development of bacterial ecology and the pathogenicity of the oral microbiota.^4^ Moreover, it acts on the biofilm structure by stopping biofilm accumulation, in addition to helping maintain pH homeostasis.^1,2,4,21-24^ Thus, arginine can be used for the prevention and treatment of caries.^4^

To the best of our knowledge, only one study has assessed the addition of arginine to orthodontic resins^6^ and no study has evaluated the addition of arginine at different concentrations. The necessity for developing antibacterial orthodontic resins combined with the thriving outcomes of arginine addition requires further studies to verify whether an increase in arginine concentration will reduce microbial count and whether the mechanical properties of the bonding materials will be affected.

Accordingly, the aim of the present study was to assess the effect of adding arginine at concentrations of 2.5%, 5%, and 7% to commercial and experimental orthodontic resins on bond strength and antimicrobial activity. The null hypotheses stated that: a) The addition of arginine at different concentrations would not interfere with the mechanical properties of the materials; b) The addition of arginine at different concentrations would not interfere with antimicrobial activity.

## Methods

The present study was approved by the local Research Ethics Committee, process no. 1038/2020.

### Study design

**Figure 1:**
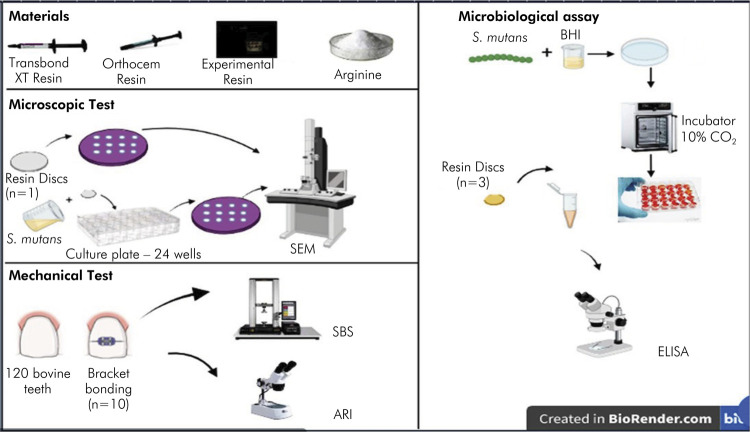
Experimental design.

The present study consisted of 12 groups: G1 – Transbond XT (TXT) commercial resin without arginine addition; G2 – OrthoCem (O) commercial resin without arginine addition; G3 – experimental resin (ER) without arginine addition; G4 – TXT with 2.5 wt.% of arginine; G5 – O with 2.5 wt.% of arginine; G6 – ER with 2.5 wt.% of arginine; G7 – TXT with 5 wt.% of arginine; G8 – O with 5 wt.% of arginine; G9 – ER with 5 wt.% of arginine; G10 – TXT with 7 wt.% of arginine; G11 – O with 7 wt.% of arginine, and G12 – ER with 7 wt.% of arginine, all of which were assessed as to their mechanical properties and antimicrobial activity (Figure 1).

### Manipulation of experimental and commercial orthodontic resins

The experimental resin (ER) used in this study was composed of bisphenol-A-glycidyl methacrylate monomers (Bis-GMA; Sigma-Aldrich Inc., St. Louis, USA) mixed with triethylene glycol dimethacrylate (TEGDMA; Sigma-Aldrich Inc., St. Louis, USA) in a 70:30 ratio (wt.%). The photoactivation system was composed of ethyl-4-(dimethylamino) benzoate (EDAB; Sigma-Aldrich) and camphorquinone (CQ; Sigma-Aldrich Inc., St. Louis, USA) at a concentration of 1 wt.% each and 0.1 wt.% BHI.^25^ Twenty-weight percent (20 wt.%) of filler particles (silanized colloidal silica – 0.04 μm) were added to the mixture (Nippon Aerosil Co. Ltd., Yokkaichi, Tokyo, Japan). The commercial orthodontic resins used were Transbond XT (3M ESPE, St Paul, USA) and OrthoCem (FGM, Joinville, Santa Catarina, Brazil) (Table 1).

**Table 1:** Materials used and application methods.

Bonding material	Composition (% weight)	Application method
Transbond TXT*	Primer: Bis-GMA and TEGDMA (45-55%), 4 – (Dimethylamino) -benzeneethanol (< 0.5%).	Active primer application for 10 s, resin application and light curing for 10 s on each side of the support.
	Resin: Bis-GMA (10-20%), Bisphenol A-dimethacrylate (5-10%), silane-treated quartz (70-80%), silane-treated silica (< 2%), diphenyliodonium hexafluorophosphate (< 1%), triphenylantimony (< 1%).	
Orthocem**	Bis-GMA, TEGDMA, methacrylic phosphate monomers, camphorquinone, tertiary amine, silicon dioxide	Application of two layers of Single Bond 2 adhesive and light curing for 10 s. Application of the resin and light curing for 10 s on each side of the support.
Experimental Resin	Bis-GMA, TEGDMA, EDAB, camphorquinone, BHI, colloidal silica.	Application of two layers of Single Bond 2 adhesive and light curing for 10 s. Application of the experimental resin and light curing for 10 s on each side of the support.
Single Bond 2 Adhesive*	Bis-GMA (10-20%), HEMA (5-15%), UDMA (< 5%), Copolymer of acrylic and Itaconic acids (5-10%), ethyl alcohol (25-35%), water (< 5%), glycerol 1,3-dimethacrylate (5-10%), diphenyliodonium hexafluorophosphate (< 0.5%), silane-treated silica (10-20%), EDMAB.	Application of two layers of Single Bond 2 adhesive, air blasting, and light curing for 10 s.

*Information from Safety Data Sheets; **Manufacturer’s information.

The total volume of each syringe material was weighed (AG 200 - GEHAKA, Ind. e Com. Eletro-Eletronica Gehaka Ltda., Sao Paulo, Brazil) under orange light and stored in dark vials to avoid its exposure to light. Thereafter, different concentrations (2.5%, 5.0%, and 7.0%) of arginine (Sigma-Aldrich Inc., St. Louis, USA) were added to commercial and experimental orthodontic resins and mechanically mixed in a centrifuge (DAC 150 Speed Mixer; Flacktek, Landrum, USA) at 3,000 rpm for 2 min, maintaining the temperature below 37°C.

### Mechanical test

#### Selection and preparation of teeth and orthodontic brackets bonding

For the adhesive analysis, 120 bovine central incisors were extracted, cleaned, and stored in 0.1% thymol aqueous solution to inhibit bacterial growth at room temperature. The teeth were placed in a PVC cylinder with chemically activated Jet® acrylic resin (Clássico, Sao Paulo, Brazil), with the buccal surface perpendicular to the horizontal axis, and then assigned randomly to 12 groups (n = 10), as described earlier, according to the resins used for bracket bonding. Before bonding, the buccal surfaces of bovine teeth were polished with pumice paste (S.S. White, Rio de Janeiro, Brazil) and deionized water using a Robson brush (Microdont, Sao Paulo, Brazil) and mounted at a contra-angle at low rotation (Kavo Dental Excellence, Joinville, Brazil) for 15 s. The Robson brush was changed after every five prophylactic procedure. Subsequently, the teeth were rinsed under running water for 10 s and dried with air jets.

Roth Light metal brackets (Morelli, Sorocaba, Brazil; Roth Prescription, maxillary right central incisor, slot 0.022”) were bonded at the middle third of the crown using the experimental and commercial resins, as described earlier. The buccal surface of the teeth was etched with 37% phosphoric acid (Condac 37 - FGM, Joinville, Brazil) for 30 s, rinsed for 15 s, and gently dried with air jets for 5 s. Prior to the placement of ER and Ortho (with or without arginine), two layers of Single Bond 2 (3M ESPE) adhesive were applied according to the manufacturer’s instructions and light-cured for 10 s with an LED Valo photopolymerized (Salt Lake City, USA) with 1,000 mW/cm^2^ irradiance, measured with a radiometer (Model 100, Demetron Research Corporation, Danbury, CT). TXT, with or without adding arginine, was applied according to the manufacturer’s instructions (Table 1). The commercial and experimental bonding agents associated with orthodontic accessories were light-cured for 10 s on each side of the bracket with an LED Valo photopolymerizer, totaling 40 s per bracket.

#### Shear bond strength (SBS) test

The tooth/bracket set was stored in an oven at 37^o^C for 24 h. After storage, the SBS test was performed on a universal testing machine (Model 4411; Instron, Canton, USA). A device was employed to align the interface of the tooth parallel to the testing device. The shear load was applied with the use of a chisel at the speed of 1 mm/min using a 50 N load cell until failure occurred. A specific software program recorded the results, and the load (in Newtons) was converted to MPa, dividing the load by the bracket area (12.3 mm^2^).

#### Adhesive remnant index (ARI)

After the SBS test, the tooth and bracket surfaces were analyzed under a light microscope (Olympus Corp, Tokyo, Japan) at 25x magnification. ARI was used to classify the failure modes (Artun and Bergland): 0 – no resin adhered to the tooth; 1 – less than half of the resin adhered to the tooth; 2 – more than half of the resin adhered to the tooth; and, 3 – all of the resin adhered to the tooth, with a distinct impression of the bracket mesh.

## Chemical and microbiological analyses

### Specimen preparation

A total of 96 resin discs (6 mm in diameter x 2 mm in height) were prepared according to group assignments (Figure 1). The discs were prepared under aseptic conditions in a laminar flow cabinet using round rubber matrices on a glass plate. After insertion of the orthodontic resin into the matrix, the specimen was covered with a polyester strip and light-cured for 40 s on an LED Valo photopolymerizer. These discs were used for the analysis of colony-forming units (CFU/mL) and for the analysis of surface morphology (SEM). The discs were sterilized under UV light using two cycles of 15 min on each side. Gram staining was performed as sterility testing of a random sample of supernatant after incubation of the discs in *Streptococcus mutans* culture.

### Colony-forming units (CFU/mL)

*Streptococcus mutans* UA159 (ATCC) strain was inoculated from frozen stocks (at -70 ^o^C) on Petri dishes containing BHI agar and incubated for 24-30 h at 37^o^C in a 10% CO_2_ atmosphere. Five colonies were transferred to test tubes with 5 mL of BHI and incubated under the same conditions for 18 h. After growth in 2-mL lidded tubes containing the resin discs (n = 6), 1 mL of each strain grown on BHI agar in the exponential growth phase (A550 nm 0.3) was added, diluted 1:10 in BHI and incubated for 18 h (37^o^C and 10% CO_2_). After incubation, the supernatant was removed, and 1 mL of sterile PBS was added to the tube, which was shaken to remove the cells adhered to the disc surface. The supernatant and PBS containing the bacterial cells removed from the disc surfaces were serially diluted, and three drops (10 µL) of each dilution on BHI agar were inoculated to determine the count of CFU/mL in each specimen (supernatant – adhered) using an Elisa spectrophotometer (VersaMax™, Molecular Devices, Sunnyvale, USA). Three independent experiments were performed, and the values were presented as means.

## Scanning electron microscopy (SEM)

### Initial surface morphology

A round specimen (6 mm in diameter x 2 mm in height) of each resin group was prepared using a polyester matrix and light-cured, as described earlier. The specimens were coated with gold/palladium (BAL-TEC SCD 050 sputter coater, Germany), and digital images were obtained at 500x magnification under a scanning electron microscope (JEOL-5600 LV, Japan) under accelerating voltage of 15 kV and working distance (WD) between 26.8 and 27.4 mm.

### Surface morphology after bacterial adhesion

Adhesive analysis and assessment of initial biofilm growth on the resin discs were performed as described earlier,^26^ with some adaptations. The discs (n = 1) were prepared and sterilized, as mentioned earlier. Each specimen was then inoculated with UA159 strains in half of the exponential growth phase at the same absorbance (A550 nm and OD equal to 0.3), and the plates were incubated at 37°C for 4 h in a 10% CO_2_ atmosphere. After incubation, the culture medium was removed with a sterile micropipette, and the discs were washed with 1.5 mL of 0.9% saline solution (NaCl 0.9%) and shaken for 15 min (Agitador MicroPlacas – MA 562, Marconi Equipamentos, Piracicaba, Brazil). The washing cycles were repeated twice. Thereafter, the discs were treated with 800 μL of 2.5% glutaraldehyde (Sigma-Aldrich) for 30 min at room temperature. The specimens were dehydrated by incubation in ethanol solutions at increasing concentrations of 50% to 100% (15 min incubation for each solution). After dehydration, the specimens were dried at room temperature and mounted on metal stubs (11x10 mm) for later gold/palladium sputtering (BAL-TEC SCD 050 sputter coater, Furstentum, Liechtenstein). Digital images were obtained at 1.000x magnification under a scanning electron microscope (JEOL-5600 LV, Tokyo, Japan) under an accelerating voltage of 20 kV and WD of 10.1 to 15.9 mm.

## Statistical analysis

The SBS findings were tested for normality (Shapiro-Wilk test) and homoscedasticity of variances (Levene’s test) before they were subjected to two-way ANOVA (resins x arginine concentrations). Multiple comparisons were made by post-hoc Tukey’s test (α = 0.05). The statistical analyses were performed using the R software (VersaMax™, Molecular Devices, Richmond, USA). The CFU/mL data were assessed by the Kruskal-Wallis test followed by Dunn’s test (α = 0.05).

## Results

### SBS test

Significant differences in SBS were observed for resin (p = 0.0001) and arginine (p = 0.006563). The double interaction between the resin and arginine was significant (p = 0.005393). Therefore, several comparisons were made using Tukey’s test for each resin and each treatment (different concentrations of arginine and no arginine). The SBS of TXT was significantly higher than that of Ortho and ER (p < 0.05) for arginine at 2.5% and 5%, but TXT and ER were statistically superior to Ortho (p < 0.05) for arginine at 7% (Table 2). No statistical difference was observed between Ortho and ER (p > 0.05) at the concentrations of 2.5% and 5% of arginine and between TXT and ER (p > 0.05) at 7%. No statistical difference was found in the untreated groups for the three resins (p > 0.05).

**Table 2:** Mean values of shear bond strength (SBS) ± standard deviation (MPa) for Ortho, TXT, and ER resins under conditions without arginine, arginine (2.5%), arginine (5%), and arginine (7%).

Orthodontic	Shear bond strength (MPa)			
Bonding Resins	Arginine 0%	Arginine 2.5 %	Arginine 5 %	Arginine 7 %
Orthocem	15.8 ± 2.3 aA	18.5 ± 3.6 bA	15.6 ± 3.4 bA	12.6 ± 3.7 bA
TXT	20.4 ± 7.2 aB	27.7 ± 9.4 aAB	29.0 ± 5.9 aA	24.1 ± 4.7 aAB
ER	12.5 ± 2.2 aB	16.3 ± 4.3 bAB	18.1 ± 5.0 bAB	21.0 ± 4.7 aA

Averages followed by lowercase distinct letters in the column and uppercase in the row differ significantly according to Tukey’s test, at the 5% level. TXT: Transbond XT; Ortho: OrthoCem; RE: Experimental resin.

Regarding ER, the mean SBS for 7% arginine was significantly higher than in the untreated groups (p < 0.05). No statistical difference was observed between arginine concentrations of 7%, 5%, and 2.5% and between the concentrations of 5% and 2.5% and the untreated groups (p > 0.05). SBS for the 5% arginine concentration was significantly higher for TXT than for the untreated groups (p < 0.05). No statistical difference was observed between the 5%, 7%, and 2.5% concentrations of arginine and between the 7% and 2.5% concentrations and the untreated groups (p > 0.05). As for the Ortho resin, there was no statistical difference between the three concentrations of arginine and the untreated groups (p > 0.05).

**Figure 2:**
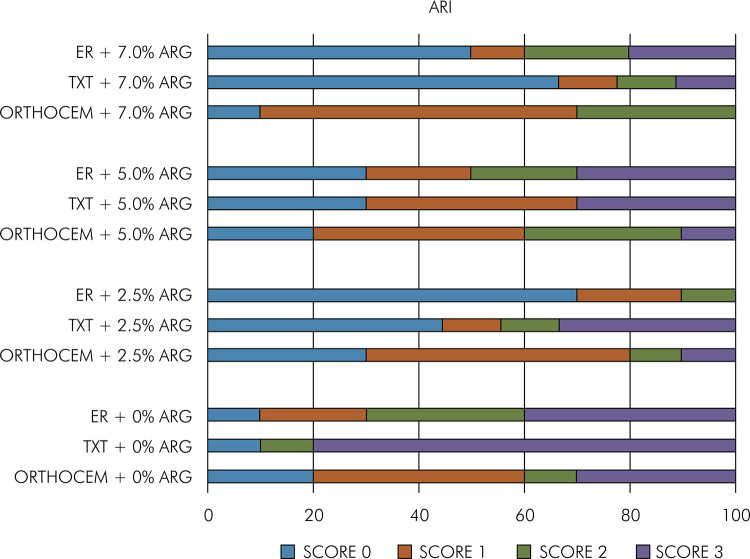
Adhesive remnant indices (ARIs).

ARI findings are shown in Figure 2. Score 3 was predominant for TXT and ER and score 1 for Ortho without adding arginine. Among the groups with the addition of 2.5% and 7% arginine, score 0 was observed for TXT and ER and score 1 for Ortho. In the groups treated with 5% arginine, score 1 was predominant in Ortho and TXT, whereas scores 0 and 3 were observed for ER.

### Chemical and microbiological analyses – *CFU/mL*

**Figure 3:**
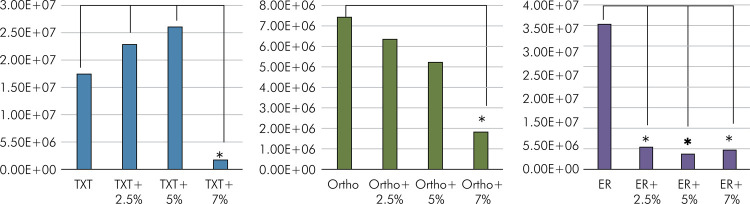
CFU/mL. The asterisk indicates statistical difference.

The addition of arginine reduced the CFU/mL of S. mutans in all assessed resins (Figure 3). The mean CFU/mL for the Ortho resin was significantly lower with the addition of 7% arginine compared to the Ortho resin without the addition of arginine (p < 0.05). No statistical difference was observed for arginine concentrations of 0%, 2.5%, and 5.0%. There was no significant difference between the Ortho resin and the Ortho resin after addition of arginine at different concentrations (p > 0.05). The addition of 7% arginine to TXT significantly reduced the mean CFU/mL when compared with the 0%, 2.5%, and 5% concentrations (p < 0.05). No statistical difference was observed between TXT without arginine and TXT with the addition of 2.5% and 5.0% of arginine (p < 0.05). All the concentrations of arginine added to ER (7%, 5%, and 2.5%) significantly reduced the CFU/mL (p < 0.05).

### Surface morphology (SEM)

**Figure 4:**
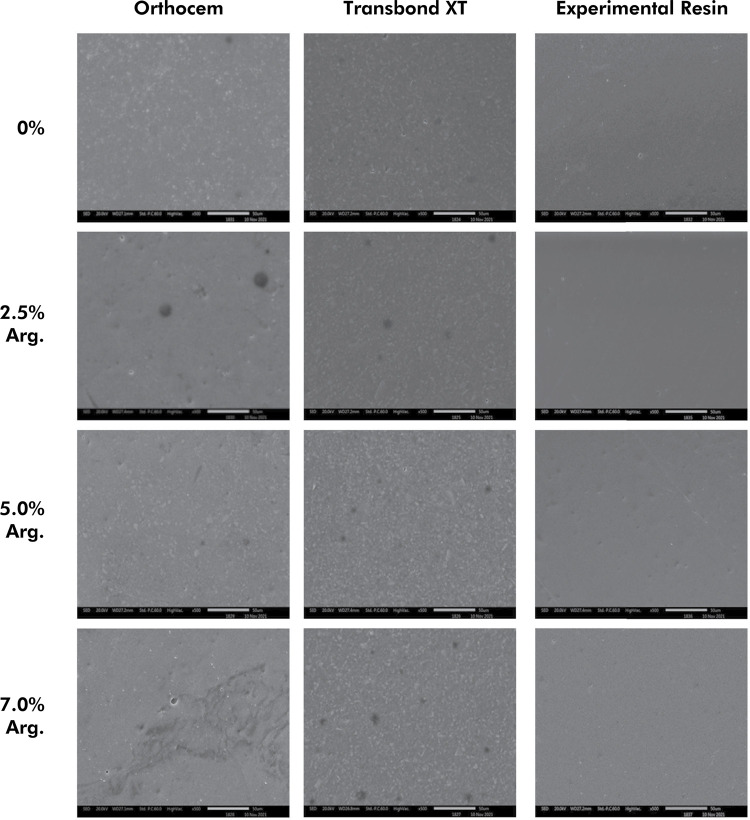
Initial surface morphology of the tested groups at 500x magnification.

**Figure 5:**
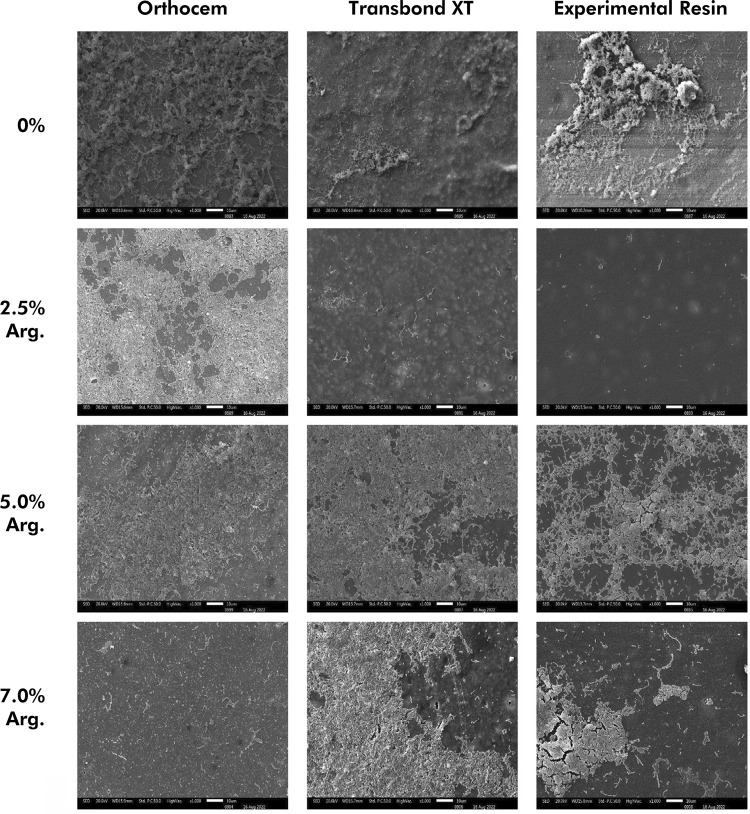
Surface morphology of the different tested groups after adhesion of S. *mutans* at 1.000x magnification.

The initial morphology of the specimens (Figure 4) and the final analysis after the adhesion of S. mutans are described in Figure 5. The initial morphological analysis, without S. mutans adhesion, showed penetration of arginine at different concentrations, regardless of the type of bonding resin (Figure 4). In the morphological analysis after S. mutans adhesion, there was a reduction in the count of bacteria adhered to the surface when compared to resins without the addition of arginine. Apparently, there was an increase in the count of S. mutans at the concentration of 2.5% of arginine for the Ortho resin and at 5% and 7% for TXT and ER (Figure 5).

## Discussion

The findings of the present study partially confirmed the null hypothesis that the addition of arginine to orthodontic resins at different concentrations would not affect SBS. Therefore, the first hypothesis was refuted. The addition of arginine to the Ortho resin did not influence SBS significantly, but arginine at different concentrations increased it significantly when added to ER and TXT, with higher SBS for TXT with the addition of 5% arginine and for ER with the addition of 7% arginine. This increase in SBS might be explained by the dissipation of the polymerization shrinkage stress of these orthodontic adhesives given that arginine was not silanized and was dispersed in the organic matrix. Note that, for all groups, the SBS found in the present study ranged from 12.6 to 29.0 MPa. It has been recommended in the literature that orthodontic adhesives have bond strengths between 5.9 and 7.8 MPa to allow retention of the bracket throughout the duration of the treatment while also allowing for of its easier removal.^14,17,18^

In general, the highest SBS in the present study was that of TXT with or without arginine, in line with previous findings.^6^ This could be explained by the presence of a higher percentage of filler particles in TXT (70% of silanized inorganic silica) when compared to Ortho (48% load) and ER (20% load). The largest number of filler particles would strengthen the orthodontic resin matrix. The lowest percentages of ARI with scores 1 and 2 for TXT, with adhesive failure, when compared to Ortho and ER, corroborate this finding (Figure 2).

In the untreated groups, there was no statistical difference in SBS between the resins. The findings of the present study are at odds with those of a previous study, in which TXT showed significantly higher SBS than that of Ortho.^6^ This could probably be explained by the application of Single Bond 2 adhesive prior to bracket bonding with Ortho and ER resins, which had a positive impact on the bond strength of these resins, as no statistical difference in SBS was found between these resins and TXT, although the literature shows that the use of adhesive prior to orthodontic adhesive has little influence on bracket retention.^27^ Applying the adhesive before the orthodontic adhesive improved penetration into the enamel micropores produced by acid etching, favoring the retention of the bracket to the tooth enamel.^28^ However, adding arginine to TXT at 2.5% and 5% showed statistically higher SBS than that of Ortho and ER resins at 2.5% and 5% of arginine. It can be inferred that the addition of arginine might have increased the viscosity of the assessed resins, reducing their penetration into the micropores created by the dissolution of the interrod enamel caused by acid etching.^29^ On the other hand, as the primer of TXT was not light-cured before resin bonding, this might have led to a greater interaction between the primer and the resin, allowing the set to infiltrate deeper into the enamel micropores.

In the case of Ortho and ER, the Single Bond 2 adhesive system was applied prior to bonding and light-cured before the placement of the resin/bracket set; therefore, the micropores might have been possibly filled by the adhesive, and that did not allow the resin to penetrate deeper, increasing its viscosity after the addition of arginine. Moreover, it is widely known that the viscosity of the adhesive is larger than that of the primer, allowing the primer to penetrate the micropores, which does not occur with the Single Bond 2 adhesive system. This is in line with previous findings,^9^ which demonstrated that when an orthodontic adhesive is viscous, a more fluid layer of resin used as a primer prior to the application of the orthodontic resin can improve the interlocking between the resin and the enamel. Furthermore, as mentioned earlier, Ortho and ER have a lower number of filler particles in their composition when compared to TXT, corroborating the ARI findings in the present study (Figure 2).

ARI has been used to show where and in what quantity orthodontic adhesive was retained after debonding. In orthodontics, the site of the bonding failure after the removal of an orthodontic bracket is important because it is necessary to treat the solid and intact enamel surface.^17^ In the groups with the addition of 5% arginine, there was a predominance of score 3 for TXT and ER, with adhesive failure at the interface between the bracket and the bonding material. The score of Ortho resin, even after the addition of arginine at different concentrations, was 1, with failures at the interface between the bonding materials and the enamel. The other resins with the addition of arginine showed a larger variation in their scores, but most had scores lower than 3. A way to minimize the risk of enamel fracture during the removal of the orthodontic bracket would be a failure at the bracket/orthodontic adhesive interface or cohesively in the orthodontic adhesive than at the bonding material/enamel interface since the adhesive residues could be removed with suitable manual or rotary instruments, in a safer way. A smaller amount of bonding material on the tooth surface indicates a shorter length of treatment.^17^

The second hypothesis – that the addition of arginine would not interfere with antimicrobial activity – was rejected. The findings showed that adding arginine to the orthodontic resins provided antimicrobial activity by reducing the growth of *S. mutans.* For the Ortho and TXT resins, only the addition of 7% arginine demonstrated a statistically significant reduction in S. *mutans* when compared to the resin without the addition of arginine. As for ER, adding 2.5% arginine was enough for a statistically significant reduction in the CFU of *S. mutans* when compared to ER without the addition of arginine.

The findings of this study partially corroborate those of a previous study,^6^ given that adding 2.5% arginine to TXT did not statistically influence CFU/mL. On the other hand, the addition of 2.5% arginine significantly influenced the reduction of bacterial load in the Ortho resin, unlike the findings of the present study. It can be assumed that this discrepancy might have occurred because of the difference in the method used for counting the CFU/mL. In this study, the bacterial count subtracted the supernatant from the adhered material. Importantly, the increase in CFU/mL in TXT with the addition of 5% arginine might have occurred because of the antimicrobial agent (arginine), which strengthened the virulence of the strain and activated resistance genes that boosted growth,^30^ but the addition of 5% arginine to TXT did not demonstrate statistical difference from TXT with and without the addition of 2.5% arginine.

In addition to the CFU/mL findings, SEM confirmed that the addition of arginine allowed reducing the growth of *S. mutans* (Figure 5). However, the increase in the number of bacteria adhered to the specimens with the addition of 2.5% arginine to the Ortho resin and 5% and 7% arginine to TXT and ER can be evaluated by the live-dead assay, which indicates the number of live bacteria on surface morphology by green staining and the number of dead bacteria by reddish staining. This would allow better identification and quantification of *S. mutans* on the surfaces of the analyzed resins. Arginine, with proven antimicrobial activity, has been widely used in dentistry (dentifrices, chews, and mouthwashes) in recent years.^4^ It neutralizes acids and modulates the pH homeostasis in the oral microbiota and also acts directly on biofilm, breaking down its structure.^1, 5,6,16,22-24^

Previous studies have suggested the presence of arginine in the oral cavity can influence the adhesion of *S. mutans* to the tooth surface, as a denser extracellular matrix was observed on a biofilm with arginine when compared to the matrix of a biofilm without arginine.^22,24^ Another study has shown that treatment with 2.5% arginine can inhibit the growth of *S. mutans* on biofilms without suppressing bacterial growth, whereas 5% and 10% arginine have more remarkable inhibition of planktonic growth and biofilm accumulation of this species.^2^ Moreover, Kolderman et al.^31^ demonstrated that L-arginine monohydrochloride inhibits bacterial growth to some extent because it breaks down the biofilm, depending on its concentration. Geraldeli et al.^16^ assessed different adhesive systems by adding arginine at different concentrations (5%, 7%, and 10%) and noted that dentin hybridization occurred properly with the addition of 7% arginine. In general, there was smaller biofilm accumulation in the presence of an adhesive with 7% arginine, which is consistent with the findings of the present study. They also observed that arginine was released at 30 days, but such release had decreased after the first 24 h.

Of note, over 50% of orthodontic treatment patients had white spot lesions, even after receiving complementary treatment with mouthwashes and fluoride varnishes.^6,11^ Accordingly, some recent studies have sought materials other than fluoride so as to allow an antimicrobial activity on orthodontic bonding resins without hindering their mechanical properties, adding different materials such as silver-doped hydroxyapatite nanoparticles;^8^ titanium dioxide nanoparticles;^15^ boron nitrate, and alkyl trimethyl ammonium bromide;^9^ methacrylate or methacrylamide monomers containing quaternary ammonium fluoride;^18^ propolis powder;^11^ and 2.5% arginine to commercial resins.^6^

Therefore, the findings of the present study seem to be very promising. The ER with addition of arginine at different concentrations used in this study and the commercial resins with 7% arginine showed antimicrobial activity without interfering with adhesive properties, indicating that arginine could be the new alternative for the prevention of white spot lesions and caries in orthodontic patients. Further studies are, however, needed to assess the effect of arginine on biofilm and its interference in the addition of experimental bonding resins, as well as its effects in the long term.

## Conclusion

The addition of different concentrations of arginine to commercial and experimental resins had a positive impact on bond strength to enamel and reduced the growth of *S. mutans* at the concentration of 7% in commercial resins and at all concentrations in the experimental resin; therefore, it may be an alternative for minimizing the development of white spot lesions without interfering with the mechanical properties of materials.
